# Molecular identification of *Bartonella bacilliformis* in ticks collected from two species of wild mammals in Madre de Dios: Peru

**DOI:** 10.1186/s13104-018-3518-z

**Published:** 2018-06-25

**Authors:** Juana del Valle-Mendoza, Jesús Rojas-Jaimes, Fernando Vásquez-Achaya, Miguel Angel Aguilar-Luis, Germán Correa-Nuñez, Wilmer Silva-Caso, Andrés G. Lescano, Xiuping Song, Qiyong Liu, Dongmei Li

**Affiliations:** 1 School of Medicine, Research and Innovation Centre of the Faculty of Health Sciences, Universidad Peruana de Ciencias Aplicadas, Lima, Peru; 2 Laboratorio de Biología Molecular, Instituto de Investigación Nutricional, Lima, Peru; 3 Laboratorio de Biología Molecular y Celular, Escuela de Medicina Humana, Universidad Científica del Sur, Lima, Peru; 4 Instituto de Investigación de Enfermedades Infecciosas, Lima, Peru; 5 Departamento Académico de Ciencias Básicas, Universidad Nacional Amazónica de Madre de Dios, Puerto Maldonado, Peru; 6 Emerge, Emerging Diseases and Climate Change Research Unit, School of Public Health and Administration, Universidad Peruana Cayetano Heredia, Lima, Peru; 7 State Key Laboratory for Infectious Disease Prevention and Control, Collaborative Innovation Center for Diagnosis and Treatment of Infectious Diseases, National Institute for Communicable Disease Control and Prevention, Chinese Center for Disease Control and Prevention (China CDC), P.O. Box5, Changping District, Beijing, 102206 People’s Republic of China

**Keywords:** *Bartonella bacilliformis*, Carrion’s disease, Ticks, Peru, PCR

## Abstract

**Objective:**

To study the presence of *Bartonella bacilliformis* in ticks collected from two wild mammals in Madre de Dios, Peru.

**Results:**

A total of 110 ticks were collected. Among the 43 *Amblyomma* spp. extracted from the 3 *Tapirus terrestris* only 3 were positive for *B. bacilliformis*. In addition, 12 out of the 67 *Rhipicephalus* (*Boophilus*) *microplus* obtained from the 3 *Pecari tajacu* were positive for *B. bacilliformis*. For the first time *B. bacilliformis* have been detected in arthropods other than *Lutzomyia* spp. Further studies are required to elucidate the possible role of ticks in the spread of South American Bartonellosis.

## Introduction

*Bartonella* species are fastidious, gram-negative bacteria associated with a wide spectrum of disease manifestations in humans and animals [1–3]. *Bartonella* spp. are organisms transmitted by a variety of bloodsucking arthropod vectors including fleas, ticks, sandflies and other insects capable of infecting humans [3, 4]. The bacteria are known to invade and replicate inside mammalian hosts erythrocytes and endothelial cells causing long-lasting bacteremia [5, 6].

South American Bartonellosis or Carrion’s disease is an infection caused by *Bartonella bacilliformis* which was first described in Peru in 1885 [1]. The infection causes an initially life-threatening phase, known as “Oroya fever”, and the second phase with chronic cutaneous manifestations called “Verruga Peruana” [3, 7]. Oroya fever is characterized by acute fever and hemolytic anemia with case fatality rates up to 88% in untreated patients. In contrast, the verruga peruana presents as angiogenic benign skin lesions which are self-limiting and can persist for 6 months [7–9]. However, most patients with Verruga Peruana do not have a clear previous history of febrile illness [7, 10].

*Bartonella bacilliformis* is known to be transmitted by the bite of the female sandfly from the genus *Lutzomyia* spp. and humans are considered the only reservoir host [3, 9]. Due to the favorable ecological conditions for the *Lutzomyia* spp., the disease has been historically restricted to remote valleys located between 500 and 3200 m (1600–10,500 feet) above sea level in the Andes Mountains of Peru, Colombia, and Ecuador [7, 9, 11]. However, in the last years, the disease has been expanding over broader geographical areas including lower altitude territories, high forest regions and valleys located in the eastern regions of the Andes representing a major health problem to populations living in endemic areas and travelers visiting these territories [12, 13].

Peru is the only endemic country with Carrion’s disease reported in 11 of its 24 departments. In addition, the disease resurgence in areas of extreme poverty has become an challenge to the country in terms of vector control and disease burden reduction [11, 14, 15]. *Lutzomyia verrucarum* is the most effective vector in Peru, with occasional outbreaks in nonendemic areas in which other species of Lutzomyias have been described, such as *L. maranonensis*, *L. robusta*, and *L. Peruensis* suggesting Lutzomyias species adaptation to different climates and heights [16–18]. However, a possible the spreading of *L. verrucarum* and other species to new areas is not sufficiently studied in Peru and a comprehensive surveillance is needed to rule out potential secondary vectors in South American regions, which may account for Carrion’s disease dissemination [19].

As new *Bartonella* spp. are discovered, the list of reservoir-adapted species and potential vectors for transmission is growing at a fast pace [20]. Furthermore, ticks have been extensively described as vectors for *B. birtlesii*, *B. henselae*, *B. grahamii*, *B. chomelii*, among other species of *Bartonella*, but not for *B. bacilliformis* yet [4, 21–23]. The aim of the present investigation is to study tick species as potential vectors for *B. bacilliformis* transmission via real-time PCR detection in ticks collected from two areas located in Madre de Dios, Peru.

## Main text

### Methods

#### Study area

The study was conducted in two areas located in the department of Madre de Dios, Peru. Both regions are in the Tahuamanu River basin, near Bolivia border territories. The collection sites are located in a forest area where wildlife hunting activity occurs. The climate is humid and warm, with average annual precipitation of 1600 mm and an average annual temperature of 25 °C.

#### Sample collection and tick species identification

The 3 Tapirs (*Tapirus terrestris*) from San Lorenzo city and the 3 Collared peccaries (*Pecari tajacu*) from Botijon town were captured for tick extraction with tweezers and then they were released with no harm. A total of 110 ticks were collected and stored individually in ethyl alcohol (96%) for preservation until they were transported to the Entomology Laboratory of the National Institute of Health “INS-Lima-Peru” for identification following the protocol published by Barros-Battesti [24]. All ticks were successfully identified and classified (Table 1).

**Table 1:** Ticks collected from San Lorenzo city and Botijon Town in Madre de Dios, Peru

Region/animal	Number of ticks (n = 110)	Lifecycle stages	Species
San Lorenzo city/*Tapirus terrestris*	14	Adult male	*Amblyomma scalpturatum*
	6	Adult female	*Amblyomma scalpturatum*
	9	Adult male	*Amblyomma naponense*
	5	Adult female	*Amblyomma naponense*
	3	Adult female	*Amblyomma latepunctatum*
	1	Adult male	*Amblyomma latepunctatum*
	3	Adult female	*Amblyomma oblongoguttatum*
	1	Adult male	*Amblyomma ovale*
	1	Adult female	*Amblyomma ovale*
Botijon Town/*Collared peccary*	22	Adult female	*Rhipicephalus* (*Boophilus*) *microplus*
	41	Adult male	*Rhipicephalus* (*Boophilus*) *microplus*
	4	Nymphs	*Rhipicephalus* (*Boophilus*) *microplus*

This study was approved by the National Committee of Health and Environment from the Regional Government of Madre de Dios.

#### DNA extraction

The ticks were processed individually, each individual was cut into pieces with the help of a scalpel and homogenized in 180 μL of PBS 1X and then the extraction of the genetic material was carried out. The DNA was extracted using the DNeasy^®^ Blood and Tissue Kit (Qiagen), according to the manufacturer’s instructions except that the final elution volume was 200 μL. The samples were stored at 4 °C until use, being thereafter stored at − 20 °C.

#### Real-time PCR assay detection of *Bartonella bacilliformis*

The qPCR was performed based on Li et al. protocol [25]. Each reaction contained 5 µL of template DNA and 15 µL of the qPCR master mix (FastStar PCR Master, Roche Diagnostic, Alemania) including 1 (10 µM) each of forward and reverse primers and 1.2 (10 µM) Taqman probe. Briefly, qPCR conditions were 95 °C for 2 min, 55 cycles of 3 s at 95 °C, 30 s at 55 °C and 10 s at 72 °C. The *B. bacilliformis* collection strain (CIP 57.19, NCTC 12135) was used as the positive control, while PCR reaction without template DNA was used as the negative control.

### Results

A total of 110 ticks were collected from both study regions. In San Lorenzo city, 43 ticks were collected from 3 *Tapirus Terrestris*; the most common species were *Amblyomma scalpturatum*, *Amblyomma naponense*, *Amblyomma latepunctatum* and *Amblyomma oblongoguttatum*. In Botijon town, 67 ticks were collected from 3 collared peccaries and all were identified as *Rhipicephalus* (*Boophilus*) *microplus* (Table 1).

All tick species were tested for the presence of *B. bacilliformis* via real-time PCR. Among the ticks collected from the *Tapirus terrestris*, only 3 were positive for *B. bacilliformis*: 2 adult *Amblyomma scalpturatum* (male and female) and 1 Adult female *Amblyomma ovale*. Among the *Rhipicephalus (Boophilus) microplus* extracted from the collared peccaries, 12 were positive for *B. bacilliformis* (4 adult females, 7 adult males, and 1 nymph) (Table 2).

**Table 2:** Ticks positives for *Bartonella bacilliformis* via Real time PCR

Region/animal	Number of ticks positive for *B. bacilliformis* (n = 15)	Lifecycle stages	Species
San Lorenzo city/*Tapirus terrestris*	1	Adult male	*Amblyomma scalpturatum*
	1	Adult female	*Amblyomma scalpturatum*
	1	Adult female	*Amblyomma ovale*
Botijon Town/Collared peccary	4	Adult female	*Rhipicephalus* (*Boophilus*) *microplus*
	7	Adult male	*Rhipicephalus* (*Boophilus*) *microplus*
	1	Nymphs	*Rhipicephalus* (*Boophilus*) *microplus*

### Discussion

*Bartonella* species are emerging zoonotic organisms capable of producing long-lasting infections transmitted by arthropods. *Bartonella bacilliformis* is the most frequent species of *Bartonella* in Peru and the responsible for Carrion’s disease a neglected tropical infection endemic in this country [3, 9].

South American Bartonellosis is an ancient illness, probably existing at least 1000 years before the arrival of Europeans since there is evidence of pre-Columbian pottery from Ecuador representing cutaneous manifestations of the disease [26]. In 1885, a Peruvian medical student named Daniel Alcides Carrion self-inoculated *B. bacilliformis* demonstrating that the two phases of illness are caused by the same bacteria and as a recognition to his sacrifice, the infection is also known as Carrion’s disease [27]. But it wasn’t until 1913 when the female sand-fly *Lutzomyia verrucarum* was confirmed as the vector responsible for its transmission to humans [11, 28].

Since the late 1990s, reports have confirmed a slow but steady increase of Carrion’s disease cases; this resurgence could be explained due to several factors including the new diagnostic tests available and climate changes which in addition to the human activities such as agriculture or hydroelectric installations may have contributed to the vector expansion to new areas [19]. Moreover, the El Niño phenomenon has created increased rainfall across the east-central and Eastern Pacific Ocean raising humidity levels favoring the sand fly’s reproduction and vector expansion [28, 29]. However, the insidious extension of the South American Bartonellosis in areas where the Lutzomyia genus is absent suggest the presence of undescribed vectors for *B. bacilliformis* [28].

Since 2004, the National Institute of Health has reported Carrion’s disease outbreaks in Madre de Dios and to date, the *L. verrucarum* is recognized as the principal vector, with other species of Lutzomyia such as *L. auraensis*, also present in the region, although these species are not vectors for the disease [30–32]. More importantly, *B. bacilliformis* infections have also been reported in territories such as San Lorenzo city and Botijon Town despite being located at 267 and 285 m.a.s.l, a much lower altitude than the 500 m.a.s.l where *Lutzomyia* spp. are commonly found. Moreover, tick-borne diseases are a significant problem in Madre de Dios and tick species, which are commonly found in *Tapirus terrestris* and *Tayassu pecari* across the territory, represent a potential vector for tropical infections [33].

In our study, we detected *B. bacilliformis* via real-time PCR in 3 *Amblyomma* spp. (*Amblyomma scalpturatum* and *Amblyomma ovale*) ticks from *Tapirus terrestris* and in 12 *Rhipicephalus (Boophilus) microplus* extracted from the collared peccaries. This is the first-time *B. bacilliformis* have been detected in an arthropod other than *Lutzomyia* spp. sand flies and could suggest that some tick species may be vectors for *B. bacilliformis.*

Polymerase chain reaction for *Bartonella* spp. isolation in arthropods is one the best available options to investigative potential vectors for these bacteria [21, 34]. However, the role of ticks in *B. bacilliformis* transmission is unknown; in contrast to other species of Bartonella which have been previously described as transmitted by ticks [4, 21–23].

In 2016, a genomic analysis on seven genera of bacteria (*Borrelia*, *Rickettsia*, *Anaplasma*, *Bartonella*, *Ehrlichia*, *Francisella* and Coxiella) described multiple pathways to mutualism for tick-borne pathogens which may confer ticks the ability to transmit new species of bacteria [35]. Unfortunately, the exact mechanisms that allow *Bartonella* to be arthropod-borne are unknown. However, it has been postulated that the ability of pathogens to be tick-transmitted may involve accumulated mutations in various genes during their evolution. Moreover, there are multiple metabolic pathways common to tick and other arthropods that may explain how bacteria adapt to new vectors, but most of them remain under investigation [36, 37].

Although the rate of evolution is greater in pathogens that are transmitted by specialized vectors, it is the rate of gene loss the phenomenon that occurs more frequently and *Bartonella* spp. are no exception. For example, *B. henselae*, a flea-borne pathogen, has a genome reduction of 12.6% in comparison to *B. quintana*, proving that this bacterium is a genomic derivate from *B. henselae* that adapted to be transmitted by louse [37]. Therefore, it is possible that other species of Bartonella such as *B. bacilliformis* might adapt to newer vectors such as ticks, but currently, there is no evidence available.

Humans are the only recognized reservoir for South American Bartonellosis and they play a significant role as perpetrators of the disease in areas of high endemicity [11]. Several reservoir candidates have been postulated for *B. bacilliformis*, from Euphorbiaceae to domestic and wild rodents; however, no study has been able to isolate the bacteria from these animals [38].

A possible explanation for *Bartonella* spp. transmission between mammals can be related to Lutzomyia species behavior as opportunistic feeders. A study conducted in an area endemic to cutaneous leishmaniasis in Lima, Peru reported that after humans the most frequent bloodmeals for *Lutzomyia peruensis* and *L. verrucarum* were cows and cats. Even though both Lutzomyias are recognized vectors for Carrion’s disease, studies are far to prove that *B. bacilliformis* can be transmitted to other mammals [39].

In conclusion, even though there are not studied mechanisms of adaptation to new vectors for *B. bacilliformis*, our results are the first ones to highlight the presence of this bacteria’s DNA in tick species. Novel studies are required to elucidate whether *B. bacilliformis* to be transmitted by ticks and the possible role of ticks in the spread of South American Bartonellosis.

## Limitations

For the first time, we have been able to isolate *B. bacilliformis* DNA in ticks from *Tapirus terrestris* and *Pecari tajacu.* However, we cannot conclude that both animals were infected or they served as a reservoir. An important limitation of this study was our inability to extract serum from these mammals for *B. bacilliformis* detection since blood collection required trained veterinary personnel.

## Abbreviations

PCR: polymerase chain reaction
DNA: deoxibonucleic acid
bp: base pairs
*B. bacilliformis*: *Bartonella bacilliformis*
PBS: phosphate buffered saline
